# Predictive value of inflammatory markers in inguinal hernia surgery: General vs spinal anesthesia

**DOI:** 10.17305/bb.2025.12262

**Published:** 2025-07-26

**Authors:** Marko Kordić, Davorin Kozomara, Ivanka Mikulić, Vinka Mikulić, Martin Kajić, Miran Boras, Mateo Bevanda, Neven Soldo, Mijo Jović, Vedran Dragišić, Ines Rozić

**Affiliations:** 1 Department of General Surgery, University Clinical Hospital Mostar, Mostar, Bosnia and Herzegovina; 2 Institute for Laboratory Diagnostics, University Clinical Hospital Mostar, Mostar, Bosnia and Herzegovina

**Keywords:** Inguinal hernia, anesthesia, lipopolysaccharide-binding protein, LBP, C-reactive protein, CRP, interleukin-6, IL-6, leukocytes

## Abstract

Inguinal hernia is a prevalent condition requiring surgical intervention, and accumulating evidence suggests that the type of anesthesia administered may influence systemic inflammatory responses. This study investigates the concentrations of inflammatory parameters in patients with inguinal hernia who underwent surgery utilizing either general or spinal anesthesia. The cohort comprised 87 male patients with inguinal hernia, classified as American Society of Anesthesiologists (ASA) physical status 1–2, who underwent elective surgical procedures. Participants were divided into two groups based on the anesthesia type: 44 received general anesthesia while 43 received spinal anesthesia. Plasma concentrations of leukocytes, C-reactive protein (CRP), interleukin-6 (IL-6), and lipopolysaccharide-binding protein (LBP) were quantified using automated immunoassays and a hematological analyzer. Standard parametric and non-parametric statistical tests were employed for data analysis, and the predictive capacity of select parameters, along with body mass index (BMI) and age, was assessed through *Receiver Operating Characteristic* (ROC) analysis with *Area Under the Curve* (AUC). Statistical analysis via the *t*-test identified significant differences in LBP concentrations (LBP 1, LBP 2, and LBP 3) between patients receiving general and spinal anesthesia. Correlation analysis of BMI and the measured parameters revealed statistically significant positive correlations for LBP 1 and LBP 2 in patients who underwent spinal anesthesia. Notably, the preoperative concentration of LBP, with a cutoff value exceeding 9.7 µg/mL, suggests a potentially superior approach with spinal anesthesia compared to general anesthesia, demonstrating 50% sensitivity and 81.4% specificity. Other parameters did not exhibit statistical significance in differentiating the type of anesthesia used for inguinal hernia surgery.

## Introduction

Inguinal hernia surgery is one of the most commonly performed surgical procedures, with approximately 20 million surgeries conducted annually worldwide [1]. Inguinal hernia operations can be performed under general, spinal, or epidural anesthesia, each of which affects the immune system differently [2]. Many immune system functions are suppressed after exposure to the combination of anesthesia and surgery [3]. Anesthesia and surgery acutely alter immune system activity through various processes in the body, predominantly involving growth factors and cytokines [4]. Cytokines are low molecular weight proteins, including interleukins, tumor necrosis factor (TNF), and interferons [5]. They are produced in activated leukocytes, fibroblasts, and endothelial cells as an early response to tissue injury and play a crucial role as mediators in inflammation and immune responses [6]. By measuring the concentration of cytokines (interleukins) in serum, it is possible to adequately assess the extent of tissue damage caused by surgical trauma and the regenerative capacity of tissues [7]. In this study, we selected lipopolysaccharide-binding protein (LBP), interleukin-6 (IL-6), C-reactive protein (CRP), and leukocytes based on their known involvement in the perioperative inflammatory response and their routine availability in our hospital laboratory. CRP and leukocytes are standard markers of acute inflammation used in surgical practice [5, 6], while IL-6 reflects early cytokine activation following tissue injury [7]. LBP, a marker of endotoxin activity and low-grade inflammation, has been associated with metabolic status and postoperative complications in surgical patients [8, 9]. The selection was also guided by cost-effectiveness and logistical feasibility.

Intraoperative modulation of surgical stress can be achieved by selecting suitable general anesthetic agents, ensuring precise fluid management, and maintaining hemodynamic stability [10]. Several perioperative factors that modulate the immune response have been identified, including different anesthetics and surgical tissue injury. However, their impact on immune system modulation may also vary depending on the context of the procedure, potentially exhibiting both pro-inflammatory and anti-inflammatory effects [11]. Through modulation of neuroendocrine, sympathetic, and inflammatory pathways, regional anesthesia attenuates the perioperative stress response, resulting in improved cardiovascular stability, reduced postoperative pain, and preserved immune competence [12]. The regulation of propofol on the function of immune cells, including lymphocytes, neutrophils, natural killer (NK) cells, and macrophages, reduces the phagocytic activity of neutrophils in innate immunity and inhibits the release of pro-inflammatory cytokines, such as IL-6 and tumor necrosis factor-α (TNF-α), from peripheral blood mononuclear cells [13]. *In vitro* studies indicate an apoptogenic effect of pancuronium on peripheral blood lymphocytes at clinically relevant concentrations, which may result in transient immunosuppression following surgical operations [14]. Kawamura et al., in a study investigating the effect of sevoflurane on cytokine secretion during coronary artery surgery, found that sevoflurane suppressed pro-inflammatory cytokines but not anti-inflammatory cytokines. This suggests that sevoflurane reduces neutrophil-dependent myocardial ischemia and reperfusion injury by suppressing inflammatory cytokines. Sevoflurane appears to be a promising and beneficial anesthetic for cardioprotection during cardiac surgical procedures [15]. The study by Kumakura et al. on the effect of nitrous oxide on the *in vivo* production of inflammatory cytokines and chemokines in the airway epithelium, in combination with sevoflurane or propofol, showed that in patients, levels of interleukin (IL)-1β, IL-8, and monocyte chemoattractant protein-1 (MCP-1) in the epithelial lining fluid (ELF) were significantly increased after surgeries involving inhalation of sevoflurane and nitrous oxide, whereas the levels of these molecules did not significantly change with inhalation of sevoflurane and air. These findings suggest that the combination of sevoflurane and nitrous oxide induces an inflammatory response and suppresses the anti-inflammatory response in the local airway environment [16]. Levobupivacaine has anti-inflammatory properties, and studies in rats have shown that it significantly protects the animals from lipopolysaccharide (LPS)-induced acute lung injury, as evidenced by a reduction in the wet-to-dry lung weight ratio, total cell count, neutrophils, macrophages, and myeloperoxidase activity, accompanied by reduced histological lung damage [17]. The immunosuppressive effect depends on the type of opioid and is independent of its potency or duration of action. Another side effect is the ability of opioids to suppress the immune response, thereby increasing susceptibility to infections. The association between opioids and immunosuppression has been investigated both *in vitro* and *in vivo*, as well as in patients. However, the results are inconsistent: exogenous opioids such as morphine and fentanyl have been shown *in vitro* and in animal studies to affect the function of macrophages, NK cells, and T-cells, and to weaken the intestinal barrier. In epidemiological studies, high doses and initiation of opioid therapy for non-malignant pain have been associated with a higher risk of infectious diseases such as pneumonia. However, clear randomized controlled trials are lacking [18]. A review of the available literature revealed that spinal anesthesia induces a smaller inflammatory response in patients undergoing total knee arthroplasty and after cesarean section [19, 20].

## Materials and methods

This study tested the hypothesis that spinal anesthesia would trigger a less inflammatory response than general anesthesia because fewer drugs that suppress the immune response are administered during spinal anesthesia. The study’s secondary objective was to examine and compare the concentrations of LBP, IL-6, CRP, and leukocytes in patients with inguinal hernias operated on under general and regional anesthesia and to determine potential correlations between these parameters. Possible outcomes arising from the stated objectives include differences in the concentrations of inflammatory markers (e.g., IL-6, CRP, LBP, and leukocytes) between the groups receiving general and spinal anesthesia. The timing and intensity of the inflammatory response after surgery were measured by serial sampling preoperatively, at 4 h, and 24 h postoperatively. Statistical significance of differences between the two groups, *P* values, confidence intervals, and correlations were assessed. Before the study, a sample size calculation was performed. Since we did not influence the choice of anesthesia type, this study can be characterized as a prospective observational study. The inclusion criteria for participants in the study were a reducible inguinal direct or indirect hernia without clinical signs of incarceration and male gender. Patients with recurrent hernia, immunodeficiency, autoimmune diseases, endocrine disorders, infectious or malignant diseases, or those undergoing corticosteroid therapy, cytostatic therapy, or radiotherapy, as well as those with an American Society of Anesthesiologists (ASA) score of 3 or higher, were excluded from the study. The day before the surgical procedure, after the patient signed the consent form to participate in the study, the patient’s height and weight were measured, and the body mass index (BMI) value was determined. Based on these values, the patients were then divided into three groups: normal weight (<25.0 BMI), overweight (25.3–30.0 BMI), and obese (>30.2 BMI).

Participants were allocated to two groups based on the type of anesthesia they received during standard clinical care. The type of anesthesia administered during the procedure was determined solely based on the clinical judgment of the anesthesiologist. Potential sources of bias, including selection bias due to anesthesia choice being determined by the anesthesiologist, were acknowledged. The same surgical technique, an open, tension-free method involving the placement of a polypropylene mesh, was applied to both groups. All procedures were performed by seven different surgeons using standardized methods and techniques to ensure consistency across cases. Blood samples were collected at three time points: 24 h before surgery (Sample 1), 4 h after surgery (Sample 2), and 24 h postoperatively (Sample 3). Serum was obtained from venous blood samples collected in tubes without anticoagulant (Sarstedt Monovette 7.5 mL), centrifuged for 10 min at 3500 rpm, and stored at –20 ^∘^C or –80 ^∘^C until analysis.

Using the fully automated ADVIA Centaur LBPS immunoassay in the ADVIA Centaur XPT system analyzer (Siemens, Dublin, Ireland), the concentrations of the following cytokines in plasma were measured: LBP using a chemiluminescent immunometric method on the solid phase from serum and IL-6 using a chemiluminescent immunochemical method from serum samples. The number of leukocytes was determined from anticoagulated human whole blood using flow cytometry with a semiconductor laser on an automated hematology analyzer (Sysmex XN 1000, Sysmex Corporation, Kobe, Japan). The concentration of CRP was determined from serum using the immunoturbidimetry method, standardized according to the International Federation of Clinical Chemistry (IFCC), on a Beckman Coulter DxC 700 AU analyzer (Beckman Coulter Inc., Brea, USA). The protocol for general anesthesia to be applied in the first group of participants is as follows: premedication - Midazolam (Dormicum) 0.1–0.4 mg/kg, Propofol 2–2.5 mg/kg, Sevoflurane MAC 0.66 (inhalational anesthesia), N_2_O 50% O_2_ 50% (inhalational anesthesia), Fentanyl 1–5 mcg/kg, Pancuronium 0.08–0.12 mg/kg, Atracurium 0.3–0.6 mg/kg. The protocol for regional anesthesia to be applied in the second group of participants is as follows: premedication - Midazolam (Dormicum) 0.1–0.4 mg/kg, Levobupivacaine (Chirocain) 10–15 mg, Fentanyl 0.025 mg (25 mcg).

Clinical parameters, including continuous electrocardiographic monitoring, heart rate, peripheral arterial oxygen saturation (using pulse oximetry), and partial pressure of carbon dioxide (using capnography), were monitored non-invasively throughout the surgery. Non-invasive systolic, diastolic, and mean arterial blood pressure measurements were performed using the intermittent oscillometric method at 5-min intervals. After the surgery, pain in patients was managed with nonsteroidal anti-inflammatory drugs (NSAIDs). No side effects, complications, or patient deaths during the hospital stay prevented sample collection.

### Ethical statement

All procedures in this study complied with the principles outlined in the Helsinki Declaration and its subsequent amendments. This study was conducted following the approval of the Ethics Committee of XXXXXX No: 1131/22 issued on August 23, 2022. Informed consent was obtained and signed manually by each patient. Inguinal hernia surgeries were performed at XXXXXX, Department of Surgery, between January 1, 2023, and December 1, 2023.

### Statistical analysis

The effect size (Cohen’s *d* index), which has empirical support in the meta-analytic study by Bakota et al., is estimated at *d* ═ 0.65 [21, 22]. The significance level (*α*) commonly used in sample size determination across previous studies was either 0.05 or 0.01; the present study will be set at 0.05 [23]. Before calculating the estimated sample size, the desired statistical power was defined as 0.90. Statistical power refers to the probability of detecting a statistically significant result using a given statistical test, assuming the effect truly exists [24]. Based on the specified parameters - effect size (*d* ═ 0.65), significance level (*α* ═ 0.05), and power (1 − β ═ 0.90) - the G*Power 3.1.7 software was used to conduct the power analysis and estimate the required sample size [25]. The study indicated a minimum sample size of 84 participants, with 42 participants per group. Additionally, the δ-index was calculated at 2.98, and the *t*-statistic value was 1.66. The Kolmogorov–Smirnov test assessed the normality of the distribution. Based on the results, statistically significant differences between data sets were analyzed using the parametric Student’s *t*-test, and correlations between the groups were examined using the Spearman rank correlation test. The significance level for all tests was set at 0.05, and *P* values less than 0.05 were considered statistically significant.

*Receiver Operating Characteristic (ROC)* analysis was performed to determine the threshold value of the observed preoperative parameters that could influence the choice of anesthesia. The *Area Under the Curve (AUC)* was calculated to evaluate the ability to differentiate between the types of anesthesia, without considering specificity and sensitivity. A statistical significance level of *P* < 0.05 was used. To verify the results of the ROC analysis, logistic regression was also estimated.

SPSS statistical software version 22 (IBM SPSS Statistics for Windows, Version 22.0, Armonk, NY, USA: IBM Corp.) was used for all analyses except for ROC and AUC, which were conducted using MedCalc^®^ Statistical Software version 22.021 (MedCalc Software Ltd, Ostend, Belgium; https://www.medcalc.org/; 2024) for the two above-mentioned analyses.

## Results

**Figure 1.**
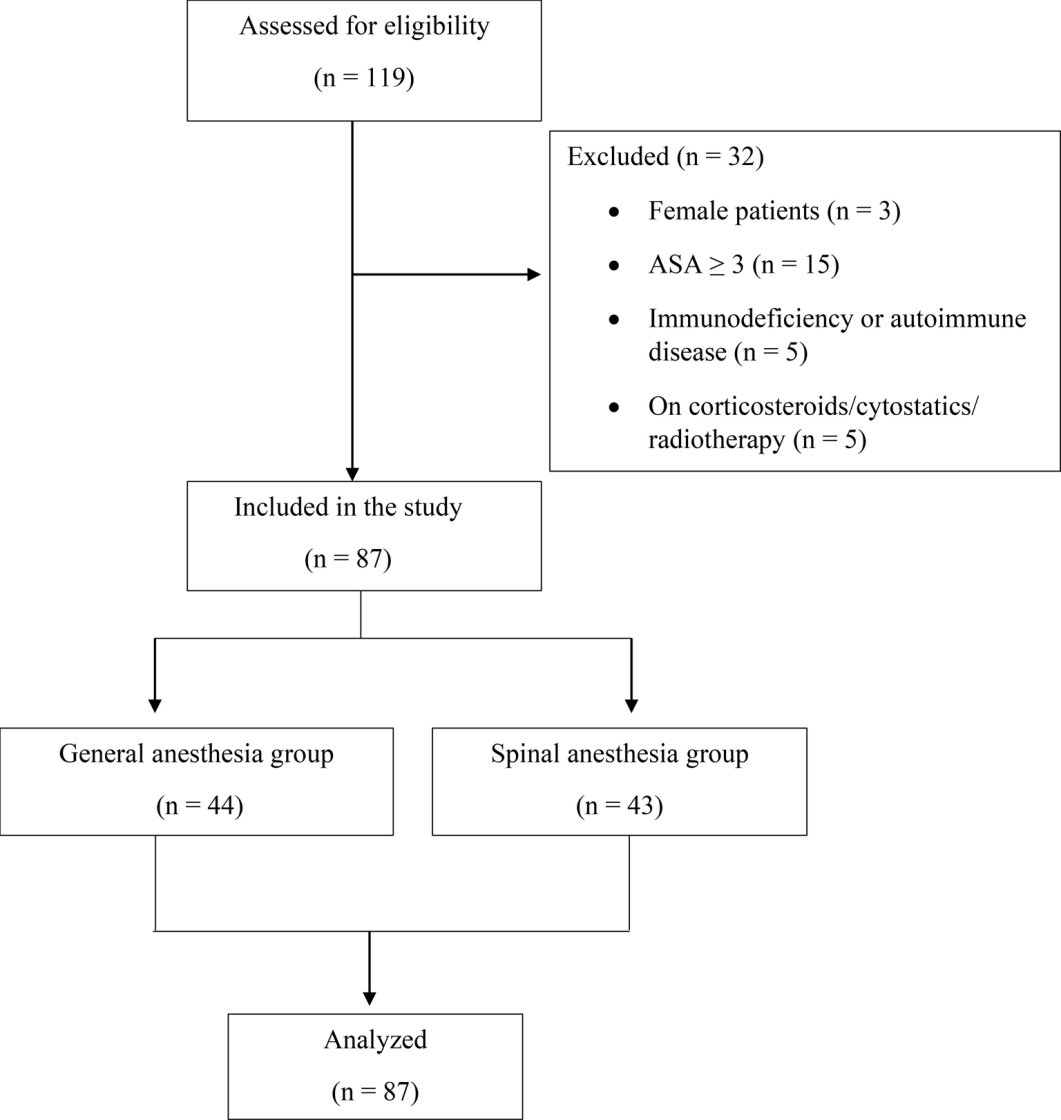
**Research flowchart.** ASA: American Society of Anesthesiologists.

The study included 87 adult male patients classified as grade 1-2 according to the ASA physical status classification system. The first group consisted of 44 patients who underwent surgery under general anesthesia, while the second group included 43 patients under regional anesthesia (Figure 1). All adult patients underwent elective inguinal hernia surgery under general or regional anesthesia. The average age of patients operated under general anesthesia was 54.9 years, with an average height of 180.84 cm and an average weight of 89.38 kg. The average age of patients operated under spinal anesthesia was 54.27 years, with an average height of 180.69 cm and an average weight of 85.79 kg. All operated patients were classified as ASA scores I and II. The average duration of the surgery was approximately 45 min (Table 1).

The results of the chi-square (χ^2^) test for the frequency of categorized BMI values indicate statistically significant differences (χ^2^ ═ 6.289, *P* ═ 0.043), with the largest difference observed in category 3 of BMI, where more patients received general anesthesia (Table 2).

**Table 1:** Patient demographic data and duration of the surgery

	**Anesthesia**										
	**General**					**Spinal**					*P*
	**Mean**	**Min**	**Max**	**Range**	**St.dev.**	**Mean**	**MIn**	**Max**	**Range**	**St.dev.**
Age	54.90	21	81	60	17.24	54.27	26	77	51	13.34	0.697
Height (cm)	180.84	160	203	43	9.07	180.69	168	196	28	6.72	0.844
Weight (kg)	89.38	55	145	90	16.42	85.79	64	116	52	9.96	0.207
BMI	2.0	1.0	3.0	2.0	0.71	1.86	1.0	3.0	2.0	0.51	0.187
Surgical duration (min)	44.77	30	65	35	9.65	45.34	30	65	35	9.47	

Patient demographic data and surgery duration: Age, height, weight, body mass index (BMI); Mean – Average value, Min – Minimum value, Max – Maximum value, Range – Range of measured values, St.dev. – Standard deviation; *P* value.

**Table 2:** Frequency of patients with three categorized BMI groups and ASA score

**Type of anesthesia**	**BMI category**			**ASA score**	
	1.0	2.0	3.0	1	2
General	11	22	11	20	24
Spinal	9	31	3	23	20
Total (N)	20	53	14	43	44

Three BMI categories based on weight gain: 1.0: Normal weight, 2.0: Overweight, and 3.0: Obese; ASA score value, ASA 1: A healthy patient; ASA 2: A patient with mild systemic disease; N: Number of patients. BMI: Body mass index; ASA: American Society of Anesthesiologists.

The Kolmogorov–Smirnov test for normality indicated that most of the observed parameters deviated from a normal distribution in the group of patients who received general anesthesia. The results of the Student’s *t*-test showed a statistically significant difference between the groups of patients who received general and spinal anesthesia for the following parameters: LBP 1, LBP 2, and LBP 3 (Table 3). Higher values for these parameters were observed in the group of patients who received general anesthesia compared to those who received spinal anesthesia.

**Table 3:** Results of the comparison of the observed parameters between groups of patients who received general and spinal anesthesia (*student t-test*)

	**Anesthesia**					
	**General**		**Spinal**		**T**	***P***
	**Mean**	**SD**	**Mean**	**SD**		
BMI	27.25	4.06	26.27	2.64	0.391	0.187
CRP 1	2.05	1.68	2.01	1.92	0.078	0.938
CRP 2	2.93	2.54	2.21	2.11	1.253	0.213
CRP 3	19.6	13.64	17.38	11.38	0.713	0.478
IL-6 1	41.97	113.7 9	14.95	84.99	1.204	0.236
IL-6 2	41.6	97.30	25.62	81.76	0.803	0.427
IL-6 3	49.32	119.2 5	25.4	93.91	1.022	0.313
LBP 1	9.44	2.68	8.21	1.97	2.508	0.0 15*
LBP 2	9.25	2.66	7.4	1.81	3.785	<0.001*
LBP 3	17.9	5.99	14.97	5.83	2.438	0.0 17*
Leuk 1	7.06	1.81	6.64	2.11	1.039	0.302
Leuk 2	10.61	2.67	10.33	2.48	0.719	0.474
Leuk 3	8.96	2.75	9.22	2.16	0.631	0.530

Continuous data are presented as the mean and standard deviation. The *t*-value is the ratio of the difference between the mean of two sets of samples to the variation that exists within the sets of samples. The *P* value represents the significance of the comparisons among the two groups. BMI: Body mass index; CRP: C-reactive protein; IL-6: Interleukin 6; LBP: Lipopolysaccharide binding protein; LEU: Leukocytes.

The CRP value in healthy individuals is less than 0.9 mg/dL. The CRP marker value above the normal range was observed in all three measurements in both patient groups, with a tendency for a more significant increase in the third measurement in both groups, more notably in the group with general anesthesia.

IL-6 values in healthy individuals range from 0–43.5 pg/mL, and values exceeding this range were only recorded in the third measurement in the general anesthesia group (49.32 pg/mL).

The LBP value in healthy individuals ranges from 5–15 µg/mL, which was exceeded only in the third measurement in the general anesthesia group (17.9 µg/mL). The normal leukocyte count in healthy individuals is between 4.5 and 11.0 × 10ˆ9 /L, so the leukocyte concentration did not exceed the allowable level in any measurement in either group.

Tables 4 and 5 present the results of the assessment of the relationship between BMI and the observed parameters in the group of patients who received general anesthesia, in the group who received spinal anesthesia, and in the total group of all patients. Since the BMI parameter deviates from a normal distribution, Spearman’s rank correlation was also evaluated.

**Table 4:** Results of Spearman’s (*rho*) correlation between BMI values and other observed parameters in general anesthesia

**Spearman’s rho**	**General anesthesia (*n* ═ 44)**
	***P***	***P***
CRP 1	−0.152	0.325
CRP 2	0.044	0.775
CRP 3	0.300*	0.048*
IL-6 1	0.184	0.232
IL-6 2	0.073	0.636
IL-6 3	0.229	0.135
LBP 1	−0.084	0.589
LBP 2	0.022	0.89
LBP 3	0.224	0.144
Leuk 1	−0.004	0.98
Leuk 2	−0.061	0.695
Leuk 3	0.082	0.595

The *P* value represents the significance of the correlations among the tested variables and the age of the patients; Spearman’s rho was used. N: Number of samples; CRP: C-reactive protein; IL-6: Interleukin 6; LBP: Lipopolysaccharide binding protein; LEU: Leukocytes.

**Table 5:** Results of Spearman’s (*rho*) correlation between BMI values and other observed parameters in spinal anesthesia

**Spearman’s rho**	**Spinal anesthesia (*n* ═ 43)**	
	***P***	***P***
CRP 1	0.155	0.32
CRP 2	0.19	0.224
CRP 3	0.203	0.193
IL-6 1	0.022	0.232
IL-6 2	0.073	0.636
IL-6 3	−0.08	0.135
LBP 1	0.491*	0.001*
LBP 2	0.334*	0.029*
LBP 3	0.024	0.879
Leuk 1	−0.106	0.5
Leuk 2	−0.004	0.982
Leuk 3	0.145	0.352

The *P* value represents the significance of the correlations among the tested variables and the age of the patients; Spearman’s rho was used. N: Number of samples; CRP: C- reactive protein; IL-6: Interleukin 6; LBP: Lipopolysaccharide binding protein; LEU: Leukocytes.

**Figure 2.**
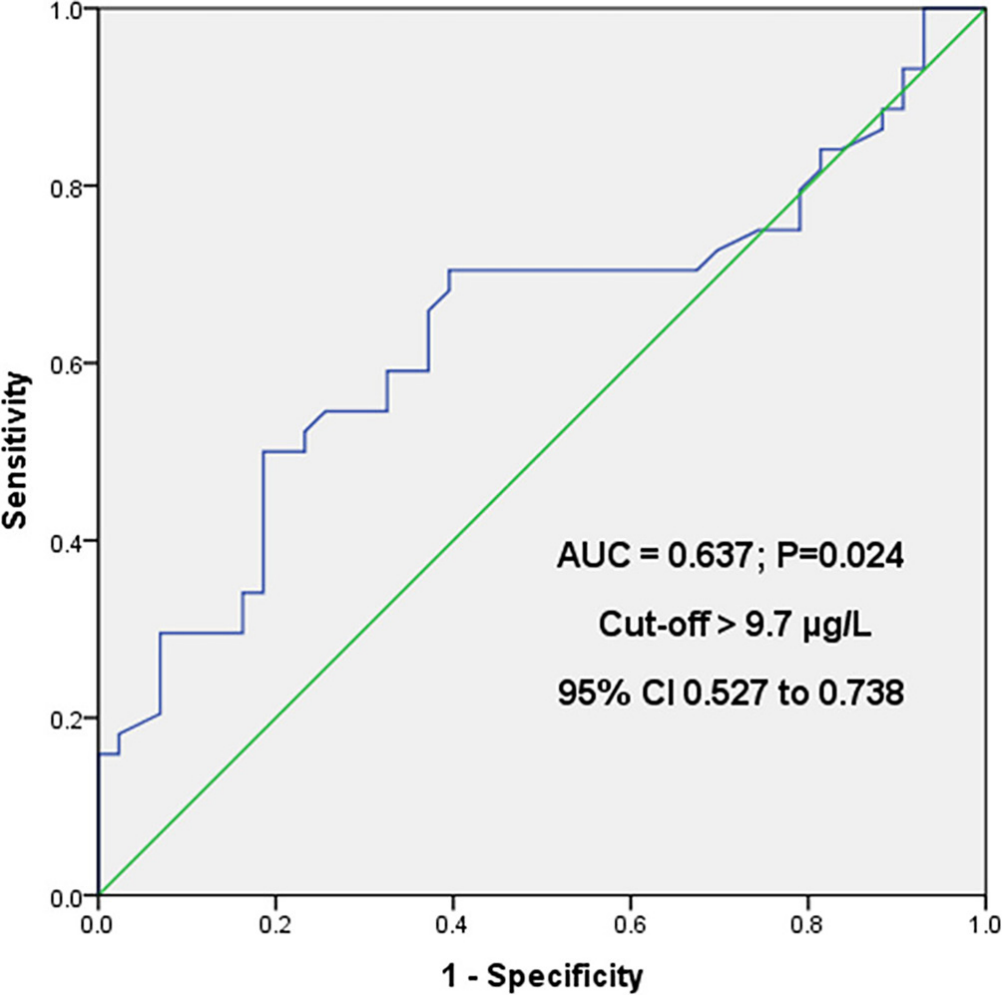
**ROC analysis with AUC for the LBP parameter before surgery (LBP 1).** ROC: Receiver operating characteristic curve; AUC: Area under the curve; LBP: Lipopolysaccharide-binding protein.

From all the analyzed combinations of BMI correlations and observed parameters, it can be concluded from Tables 3 and 4 that statistically significant positive correlations were observed for the LBP 1 and LBP 2 parameters in the group of patients who received spinal anesthesia. For general anesthesia, a statistically significant moderate positive correlation was found between BMI and CRP 3. To further investigate the potential of some of the observed parameters measured before surgery, as well as BMI and age, ROC analysis with AUC was performed. The preoperative LBP parameter showed that a value (cut-off) over 9.7 µg/mL indicates a potentially better outcome for spinal anesthesia compared to general anesthesia, with 50% sensitivity and 81.4% specificity (AUC = 0.637, *z* ═ 2.250, *P* ═ 0.0244) (Figure 2).

The moderate sensitivity and specificity value enable a solid assessment of LBP’s limit (cut-off) value.

## Discussion

To the best of our knowledge, this study is the first to provide information on the dynamics of the inflammatory response based on the type of anesthesia used in patients undergoing inguinal hernia surgery. We demonstrated that the preoperative value of the LBP parameter may indicate a potentially better approach with spinal anesthesia compared to general anesthesia.

Most of the immune system’s actions and the acute inflammatory response to exogenous infections become evident after surgery. Disrupting the skin, tissue, or organ barriers triggers an immune response with the release of inflammatory mediators [26]. Anesthesia affects the immune response through central modulation in general anesthesia, afferent blockade in regional anesthesia, and interaction with the endocrine system. Anesthetics have a suppressive effect on cellular and humoral immunity by acting on the function of immune-competent cells and influencing the gene expression of inflammatory mediators and their secretion [27]. Assessments of the effects of anesthetics on the immune system in patients are quite limited due to several variables, such as the duration and type of surgical procedure and related complications [28]. Regional anesthesia, either alone or in combination with general anesthesia, can significantly suppress the body’s endocrine response to stress [29]. Regional anesthesia performed in laparoscopic inguinal hernia surgeries yields similar results and complication rates as general anesthesia but results in less postoperative pain [30]. In a meta-analysis of six randomized controlled trials and five cohort studies, Li et al. found that spinal anesthesia can be another good option for pain relief, regardless of whether the surgery is open or laparoscopic for hernia repair. However, they also concluded that spinal anesthesia could not be proven to be superior to general anesthesia [31]. Wongyingsin et al., in a randomized clinical study of postoperative clinical outcomes and inflammatory markers following inguinal hernia surgery with local, spinal, and general anesthesia, observed no statistically significant differences between the groups in terms of postoperative pain intensity, length of hospital stay, complications, or changes in inflammatory markers [8]. LPS is a component of the outer membrane of gram-negative bacteria, and there is increasing evidence that LPS could be a key factor in low-grade inflammation associated with obesity [9]. In the group of patients who underwent surgery under general anesthesia, there was a higher number of patients classified into the BMI 3.0 group (3:11), and consequently, a significantly higher number of patients with a higher BMI. This may explain the increased level of LBP 1 (before surgery) in the general anesthesia group (9.44 µg/mL) compared to the spinal anesthesia group (8.21 µg/mL) (Table 2). The results of Spearman’s correlation show a statistically significant positive correlation for the LBP 1 and LBP 2 parameters, with LBP 1 and LBP 2 values correlating with BMI decreasing in the spinal anesthesia group. These results align with the findings of Watanabe et al., who determined that plasma LBP concentrations are significantly associated with BMI and serum levels of high-sensitivity hs-CRP as BMI increases in patients in Japan [32]. It is believed that the increase in CRP levels in obesity is due to the rise in fat tissue and the accumulation of free fatty acids, which stimulate the release of pro-inflammatory cytokines and CRP synthesis in the liver [33]. It was observed that CRP 3 was moderately positively correlated with BMI in the group of patients who received general anesthesia. The moderate increase in CRP can be explained by the anti-inflammatory effects of general anesthesia, considering that the patients in the general anesthesia group had slightly higher BMI. No increase in CRP was observed in the patients who received spinal anesthesia, which suggests that spinal anesthesia may be a better option for inguinal hernia surgery, particularly in patients with a BMI greater than normal (>25). The AUC ROC analysis of the LBP parameter before the surgical procedure showed that a value over 9.7 µg/mL indicates a potentially better approach with spinal anesthesia compared to general anesthesia, with 50% sensitivity and 81.4% specificity (AUC = 0.637, *z* ═ 2.250, *P* ═ 0.0244) (Figure 2). The moderate sensitivity and specificity values provide a solid estimate of the cut-off value for LBP. The significance of this study lies in its focus on one of the most common surgical interventions and its potential prognostic value for the preoperative LBP level, which could help determine the type of anesthesia that is more favorable for the patient and reduce the risk of inflammation.

### Study limitations

Part of the funding for this research was provided by the management of the University Clinical Hospital, Mostar, while the remaining costs were covered by the authors personally. The scope of the study was limited by the necessity to define a hypothesis and preselect inflammatory markers at the outset. The choice of LBP, IL-6, CRP, and leukocytes was guided by their clinical relevance, established role in postoperative immune response, and routine availability in our hospital laboratory. Other markers, such as TNF-α, IL-1β, or procalcitonin, were not included due to limited financial resources and logistical feasibility. Future studies with expanded budgets and access to broader biomarker panels may provide more comprehensive insights. Another limitation of this study is the exclusion of female patients with inguinal hernia. Although three female patients underwent surgery during the study period, the sample size was insufficient for statistical analysis, and they were therefore not included in the final dataset. Further studies including female patients are necessary to improve the generalizability of our findings. Additionally, due to the short postoperative hospital stay, we were only able to monitor inflammatory markers for 48 h post-surgery. Future research should aim to include larger cohorts, extend the duration of follow-up, and apply more robust statistical techniques to validate these findings. Other unmeasured variables, such as smoking, alcohol use, medication history, and undiagnosed comorbidities, may also have influenced the results.

## Conclusion

The statistical analysis of the obtained data on the values of the inflammatory parameters demonstrates that an LBP value of over 9.7 µg/mL before surgery suggests a potentially better approach with spinal anesthesia compared to general anesthesia, with 50% sensitivity and 81.4% specificity (AUC = 0.637, *z* ═ 2.250, *P* ═ 0.0244). The moderate sensitivity and specificity values allow for a reliable assessment of the LBP cut-off value. The study also identified an association between higher BMI and LBP levels before and after surgery, as well as a positive correlation between BMI and CRP levels. Other inflammatory parameters did not show statistically significant differences in determining the appropriate type of anesthesia for inguinal hernia surgery.
